# Completion of maternal and child health continuum of care and associated factors among women in Gode district, Shebele Zone, Eastern Ethiopia, 2022

**DOI:** 10.1186/s12884-024-06639-0

**Published:** 2024-06-24

**Authors:** Liyew Mekonen Ayehubizu, Semehal Haile Yohannes, Zemenu Shiferaw Yadeta, Metsihet Tariku Fetene

**Affiliations:** 1 Department of Public Health, College of Medicine and Health sciences, Jigjiga University, Jigjiga, Ethiopia; 2 Department of Neonatal Nursing, College of Medicine and Health sciences, Jigjiga University, Jigjiga, Ethiopia; 3 Department of Reproductive Health and Population Studies, School of Public Health, College of Medicine and Health Science, Bahir Dar University, Bahir Dar, Ethiopia; 4 Department of Nursing, College of Medicine and Health sciences, Jigjiga University, Jigjiga, Ethiopia

**Keywords:** Maternal and child health continuum of care, Completion & associated factors

## Abstract

**Background:**

The Continuum of care for reproductive, maternal, newborn, and child health includes integrated service delivery for mothers and children from pre-pregnancy to delivery, the immediate postnatal period, and childhood. In Ethiopia, the magnitude of antenatal care, skilled delivery, postnatal care, and immunization for children have shown improvement. Despite this, there was limited research on the percentage of mothers who have completed maternal and child continuum care.

**Objective:**

To assess the Completion of Maternal and Child Health Continuum of Care and Associated Factors among women in Gode District, Shebele Zone, Eastern Ethiopia ,2022.

**Method:**

A community-based cross-sectional study design applied from November 1–15, 2022. A stratified sampling method was applied. A woman who had two 14–24 months child preceding the data collection period were included in the study. An interviewer-administered semi-structured questioner had been used for data collection. Data collected by using kobo collect and analyzed using STATA version 17. Both Bivariable and multivariable logistic regression analyses were done. In multivariable analysis, variables having P-values ≤ 0.05 were taken as factors associated with the completion of the maternal and child health continuum of care.

**Result:**

The Completion of maternal and child continuum of care was 13.5% (10.7-17.0%) in Gode district,2022. Accordingly, Husband occupation (Government employee) [AOR = 2.3, 95%CI 1.2–4.7] and perceived time to reach health facility (less than 30 min) [AOR = 2.96, 95%CI 1.2–7.5] were factors showing significant association with maternal and child health continuum of care among mothers in Gode district, Somali regional State;2022 at P-value ≤ 0.05.

**Conclusion and recommendation:**

Only 13.5% of mothers in Gode district received all of the recommended maternal and child health services during their pregnancy, childbirth, and postpartum period. The study found that two factors were associated with a higher likelihood of receiving Maternal and child continuum of care: Government employed husband and perceived time to reach a health facility. Governments can play a key role in increasing the maternal and child health continuum of care by investing by making health care facility accessible.

## Introduction

The Continuum of Care for reproductive, maternal, newborn, and child health (RMNCH) includes integrated service delivery for mothers and children from pre-pregnancy to delivery, the immediate postnatal period, and childhood [1]. Continuum of care(COC) is one of the important long-term plans for reducing maternal and neonatal deaths and improving the health and well-being of mothers and newborns [2, 3].

Despite global advocacy by the World Health Organization (WHO) and its allies for a continuum of care (CoC) approach to improve maternal, newborn, and child health (MNCH) outcomes, with policies like Ethiopia’s emphasizing staged, evidence-based interventions [4–7], significant gaps remain [1, 8]. This is a critical issue as the world strives to drastically reduce maternal and child deaths by 2030. Achieving this ambitious goal, with targets like lowering maternal mortality from 216 to less than 70 per 100,000 live births, hinges on ensuring accessible, high-quality care throughout pregnancy, childbirth, and the postpartum period [9]. The current disparity in CoC completion highlights the need for further efforts to bridge this gap and optimize maternal and child health outcomes globally [10, 11].

While global statistics show a high proportion of women accessing antenatal care at least once (86%), only two-thirds complete the recommended minimum of four visits [12]. This disparity is particularly stark in regions with high maternal mortality rates, such as Sub-Saharan Africa and South Asia, where even fewer women receive adequate prenatal care and deliver in health facilities [12]. Despite some improvements in Ethiopia, evidenced by increased utilization of various maternal health services (32–43% for prenatal care, 28–50% for skilled delivery assistance, 17–34% for postnatal care, and 39–43% for full childhood immunization), the gap in completing the entire continuum of care remains significant, highlighting the need for further progress in low and middle-income countries [13–16].

The maternity continuum of care has a plethora of advantages; that is why different scholars tried to address the issue and factors associated with it. The overall literature review; ranges from 60% in Cambodia to 27% in Pakistan [17, 18]. In Africa, it ranges from 8% in Ghana to 31% in Nigeria [4, 19]. According to a study done in Ethiopia, the magnitude of maternal, newborn, and child health continuum of care in 2019 was 9.1% [20].

The respondent’s age, educational status, essential ANC components, occupational status, parity, women who planned for pregnancy, mothers who use contraceptives before pregnancy, and wealth status were the factors associated with the maternity continuum of care [21–23]. But some variables require further investigation.

There is a significant drop in maternal and child health continuum of care and still, little progress has been made in closing the gap between maternal and child health services [24]. This lack of quality of care reflects lost opportunities and a risk factor for adverse results in maternal and child health [25]. Significant gaps exist in achieving the recommended continuum of maternal healthcare services across Ethiopia, including the Gode district. This lack of research on completion rates and the factors influencing them in Gode specifically could hinder the development of effective, context-based interventions that are crucial for reducing maternal and child mortality.

## Methods

### Study area and period

This study was conducted in Gode district, Shebelle Zone, Ethiopia, from November 1–15, 2022. Based on 2022 population projections, Gode district has over 163,894 residents with a nearly even gender distribution (45.6% male and 54.4% female). The district encompasses an area of 7,700 square kilometers and experiences a 2.7% annual population growth.

Gode city, the main town in the district, has limited healthcare facilities. These include 2 health centers, 1 general hospital, and 4 health posts. Gode’s location is roughly 1225 km from Addis Ababa, Ethiopia’s capital city, and 600 km from Jigjiga, the capital of the Somali Regional State.

### Study design

A community-based cross-sectional study design was employed.

### Population

#### Source population

All women who had 14–24 months child preceding the data collection period in Gode district, Shebelle zone, Eastern Ethiopia.

#### Study population

Randomly selected women who had 14–24 months child preceding the data collection period in Gode district, Shebelle zone, Eastern Ethiopia.

### Inclusion and exclusion criteria

#### Inclusion criteria

Women whose child is alive at the time of data collection.

#### Exclusion criteria

Mothers are unable to communicate due to severe illness, limiting the speaking and responding activity of the respondents at the time of data collection.

### Sample size calculation

Researchers initially calculated a sample size using the single population proportion formula. This method assumed a 5% margin of error, a 95% confidence level, and a 50% completion rate for the maternal and childcare continuum. To account for potential participants who wouldn’t respond, a 20% non-response rate was added, resulting in a sample size of 461.

However, to explore factors potentially influencing MCH continuum completion, researchers employed the double population proportion formula. By applying the following assumptions: a 95% confidence level, 80% power, and a roughly equal number of participants in groups with and without the factor being investigated. The formula was applied to various factors identified from existing literatures: Exposure to media (*n* = 152), Place of residence (*n* = 189), Birth preparedness and complication readiness (*n* = 222), Wealth index (*n* = 152), Danger signs of pregnancy (sample *n* = 114), using Epi Info software version 7 (Table 1).

**Table 1:** Sample size calculation using double population proportion formula by considering variables associated with maternal and child health continuum of care

Variables	% Outcome in unexposed group	AOR	CI	Power	Sample size	Final sample size including non-response rate	Reference
Exposure to media	68.1	2.62	95	80	138	152	(27)
Place of residence	54.8	2.07	95	80	172	189	(21)
Birth preparedness and complication redness	6.64	2.93	95	80	202	222	(28)
Wealth index	44.6	2.21	95	80	138	152	(24)
Danger sign of pregnancy	69.5	3.31	95	80	104	114	(24)

However, the sample size calculated using single population proportion formula was higher than the sample sizes obtained when using the double population proportion formula for specific factors. So, the final sample size was 461.

### Sampling procedure

A stratified sampling technique was used. In the initial stage, Kebeles was discovered in the Gode district, which was divided into urban and rural areas. Then, A random sampling methodology, specifically a lottery, was employed to select six kebeles. For selected kebeles, the determined sample size is distributed proportionally. Before data collection, a list of women who gave birth in the previous year was gathered from kebeles, as well as the delivery and immunization registration book of health extension workers Finally, each study participant was chosen at random.

From a total 946 mothers who had a child 14–24 months. Within Gode city, a sample of six kebeles was drawn: Kebele Two (*n* = 53), Kebele Five (*n* = 33), Kebele Six (*n* = 31), Kebele Seven (*n* = 57), Kebele Eleven (*n* = 43), and Kebele Thirteen (*n* = 51). Four additional kebeles were selected from rural areas: Hadhawe (*n* = 60), Ilan (*n* = 55), Badila’ad (*n* = 40), and Barsan (*n* = 38).

### Data collection tool and procedure

The questionnaire was prepared in English and translated into Somali language. The questioner incorporates socio-demographic, household-related, community, reproductive and obstetric, and institutional factors. The tool was first pretested in the Kebridhar district. Data collected by a semi-structured questionnaire through face-to-face interviews using a kobo collect toolbox. Ten midwives for data collection and four public health officers with a bachelor’s degree as supervisors were involved. The data collectors and supervisors got training for one day about the objective of the study, study design, and the content of the questionnaires.

### Study variables

#### Dependent variable

Maternal and child health continuum of care (completed, not completed).

#### Independent variable

**Socio-Demographic Characteristics.** age, marital status, mother’s educational status, mother’s occupation, and husband’s occupation.

**Reproductive and Obstetric factors.** Parity of women, age at 1st birth, planned Pregnancy, pre-pregnancy contraceptive utilization, birth preparedness, and complication readiness, preconception contraceptives utilization, the timing of ANC, knowledge on key danger signs of pregnancy, delivery and postpartum and mode of delivery.

**Households related factors.** Wealth index, information on maternal and child health services, Decision-making autonomy.

**Community factors.** means of transportation to the health facility, distance from the district hospital, place of residence.

**Institutional related factors.** the behavior of health professionals, satisfaction with service delivery, and availability of drug and medical equipment.

### Data quality control

The questionnaire was first prepared in English and then translated to Somali language and finally back translated to English by different individuals to check its accuracy and consistency. Before data collection, the aim of the study was told to the study participants. Intensive training has been given to data collectors and supervisors. The questionnaire was pretested outside the study area at Kebridhar district with 5% of the sample size to check the language clarity and appropriateness of the questionnaire, and corrections was taken. The data collection process was supervised daily, and each questionnaire was checked for completeness.

### Operational definition and definition of terms

**Maternal and Child Health Continuum of Care.** A woman and her child were considered having completed the continuum of care when a woman; had four or more ANC visits by skilled health personnel (medical doctors, midwives, nurses, health officers) and childbirth by SBA at health institution and PNC at least once after discharge from the health facilities within six weeks by skilled health personnel at the health facility or with in the first week by community health extension workers during their home visit, and her child completed the immunization. Otherwise, a woman-child pair is not considered or classified as completed the continuum of care if they miss any one of the above visits or attendance at any level of care [26, 27].

**Fully Vaccinated Child.** A child is considered fully vaccinated when a child receives one dose of BCG, three doses of pentavalent, Pneumococcal Conjugate (PCV), Oral Polio Vaccines (OPV), two doses of Rotavirus, and one dose of measles vaccines [28].

**Not Immunized.** A child aged 14–24 months who did not receive any vaccine before this study is considered not immunized [27].

### Data processing and analysis

Data collected by using kobo collect and exported to Stata 17 for analysis. Descriptive statistics was done to quantify the proportion of women who complete the continuum of care for maternal and child health services. Findings summarized in tables and graphs using frequencies and percentages.

Initially, Bivariable logistic regression analysis was performed between the dependent variable and each of the independent variables in sequence. Variables having a p-value of < 0.20 in Bivariable logistic regression selected as candidates for multivariable logistic regression analysis. Model fitness was checked by Hosmer and Lemeshow goodness of test and multicollinearity between the explanatory variables checked using a variance inflation factor. Association between an outcome variable and explanatory variables are reported by using adjusted odds ratio and its 95% CI, and variables having p-values ≤ 0.05 in the multivariable logistic regression model were considered as statistically significant.

### Ethical clearance

Ethical clearances with this reference number (RERC/075/2022) obtained from the Ethics research review committee of Jigjiga University and further approval gained from Shebelle zone health office and the purpose or aim of the study be briefly explained for the respondents and written informed consent obtained from each respondent. We obtained parental permission for all participants under the age of 18, as is standard practice for research involving minors and assent from them.

During data collection, we assured participants that all information collected would be kept anonymous and confidential. Mothers had the right to withdraw from the study at any point or decline participation altogether. Their involvement was entirely voluntary.

## Result

### Sociodemographic characteristics

Out of 461 samples a total of 459 mothers were interviewed making a response rate of 99.6%. Regarding an age, 86 (17. %) of the women were under 24 years old, while 210 (45.6%) were between 25 and 34 years old. The mean age with standard deviation of the participant was 31.4(± 7.5) years. The study participants were predominantly Somali 454(98.9%) and Muslim 456 (99.4%). Most were married 433 (95.6%) and lacked formal education 236(51.5%). In most households (92.2%), the husband was the head and breadwinner 307 (66.9%). The average family size was 5.9, with 262(85.6%) having two children under five (Table 2).

**Table 2:** Sociodemographic characteristics of women in Gode districts, Somali regional state; Ethiopia;2022

Variables	Category	Frequency	Percentage
Age of women in years	15–24	86	18.7
	25–34	210	45.8
	35–49	163	35.5
Ethnicity	Somali	454	98.9
	Amhara	3	0.7
	Oromo	2	0.4
Religion	Muslim	456	99.4
	Orthodox	2	0.4
	Protestant	1	0.2
Marital Status	Married	433	94.3
	Widowed	7	1.5
	Divorced	19	4.1
Educational Status	No Education (Unable to read and write)	236	51.4
	Primary Education (1–8)	173	37.7
	Secondary and above	50	10.9
Maternal Occupation	Government Employee	116	25.3
	Marchant	85	18.5
	Farmer	80	17.4
	Housewife	178	38.8
Husband Occupation	Government Employee	167	37.0
	Farmer	135	29.9
	Marchant	71	15.7
	Others^1^	78	17.3
Head of the household	Husband	423	92.2
	Wife	36	7.8
Bread Winner	Husband	307	66.9
	Wife	38	8.3
	Both	114	24.8
Family Size	Less than five	246	53.6
	Six up to Ten	186	40.5
	Eleven up to Fifteen	27	5.9
Under five children	One	131	28.5
	Two	262	85.6
	Three	66	14.4
Family income (In Birr)	< 5,000	147	32.0
	5,000–10,000	170	37.0
	10,000–20,000	111	24.2
	> 20,000	31	6.8

### MCH continuum of care

**Fig. 1:**
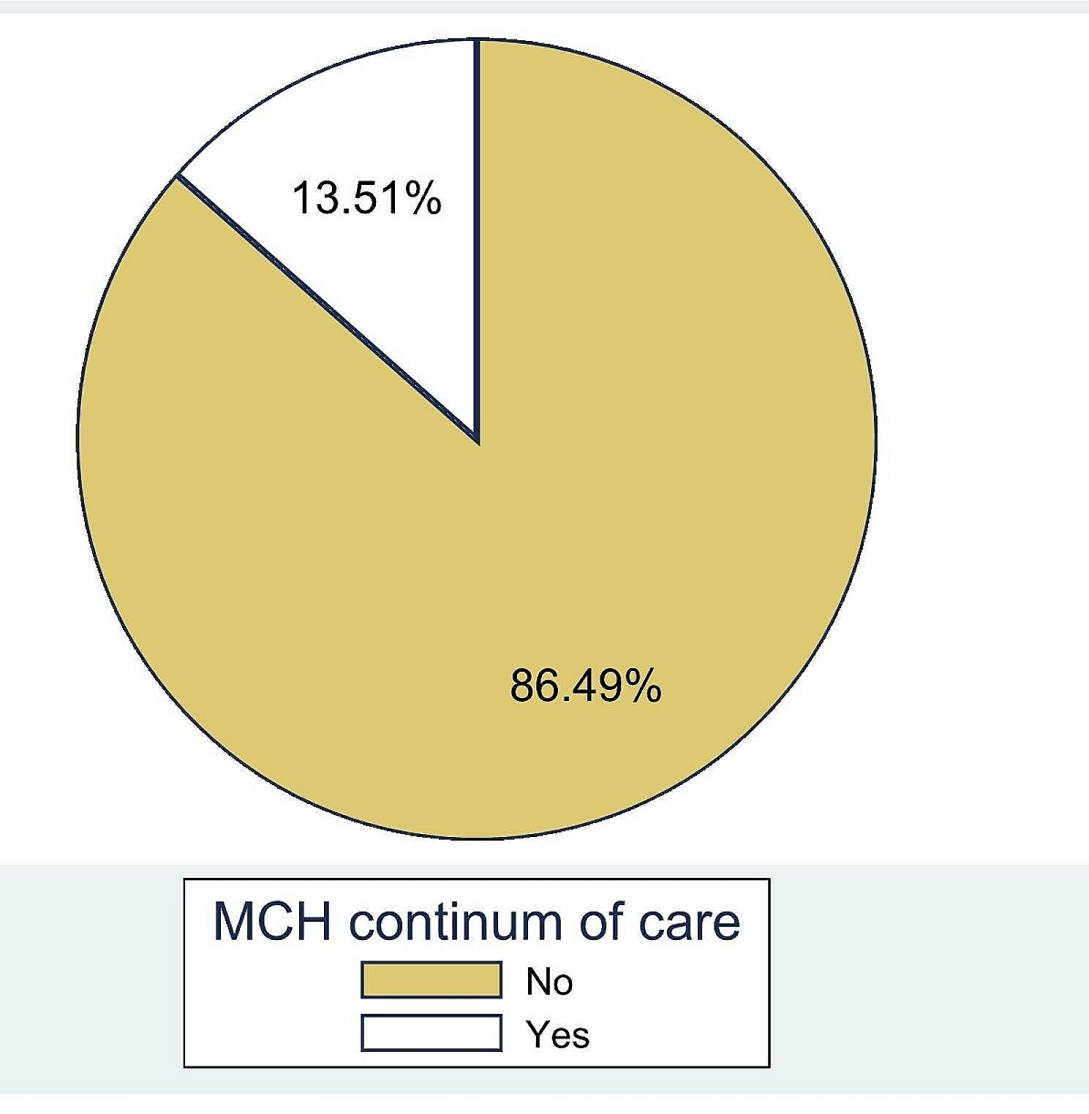
Prevalence of Maternal and child health continuum of care in Gode district, Somali Regional State;2022

A high proportion of women (90.9%) reported attending ANC visits during their previous pregnancy. The distribution of antenatal care (ANC) visits varied, with nearly a fifth (19.2%) of women attending four or more times. The majority of women (88.7%) delivered at a health facility. Encouragingly, a large majority (81.9%) reported attending postnatal care (PNC) services, though attendance frequency varied with one-third attending only once (34.4%). Three-quarters (74.3%) of children had complete or up-to-date immunizations (Table 3). Overall, the prevalence of the maternal and child health (MCH) continuum of care in Gode district, 2022 was 13.5% (10.7-17.0%) (Fig. 1).

**Table 3:** Maternal and child health service utilization in Gode District, Somali Region, Ethiopia, 2022

Variables	Category	Frequency	Percentage
ANC in previous pregnancy	Yes	417	90.9
	No	42	9.2
Number of ANC visit	Once	83	19.9
	Twice	137	32.9
	Three	117	28.1
	Four and more	80	19.2
Place of delivery	Health facility	407	88.7
	Home	52	11.3
PNC attendance	Yes	376	81.9
	No	83	18.1
Time to Attended PNC	One time	142	34.4
	Two time	138	33.4
	Three time	62	15.0
	Four and more	71	17.2
Immunization up-to-date or complete	Yes	341	74.3
	No	118	25.7
MCH continuum of care	Complete	62	13.5%
	Incomplete	397	86.5%

### Factors associated with maternal and child health continuum of care

#### Sociodemographic characteristics with MCHCC

Crude analysis of socio-demographic characteristics through bivariate logistic regression showed that age of women 25–34 years (COR = 2.4, 95% CI = 0.96–5.96, p-value = 0.06), (COR = 2.3, 95% CI = 0.9–5.9, p-value = 0.08), Ethnicity(Somali) (COR = 0.1,95%CI = 0.02–0.6, p value = 0.0012), Religion (Muslim) (COR = 0.3, 95% CI = 0.03–3.5, p-value = 0.340), marital status(married) (COR = 0.6, 95%CI = 0.2–1.8, p-value = 0.383), Educational status(Primary education 1–8 grade) (COR = 2.0, 95%CI = 1.1–3.5, p-value = 0.017), Maternal occupation Government employee (COR = 1.6, 95%CI = 0.9–2.9, p-value = 0.096), Husband occupation(Government employee (COR = 1.7, 95% CI = 0.99–2.96, p-value = 0.053), head of the house hold (husband) (COR = 0.96, 95% CI = 0.36–2.59, p-value = 0.944), Bread winner wife (COR = 2.0, 95% CI = 0.8–4.9, p-value = 0.128) & both (COR = 2.4, 95% CI = 1.3–4.3, p-value = 0.004), Family size(6-100) (CI = 1.5, 95% CI = 0.9–2.6, p-value = 0.154), Under five children (3) (CI = 2.7, 95% CI = 1.2-6.0, p-value = 0.154) and family income in birr (5000–10,000) (CI = 1.7, 95% CI = 0.8–3.3, p-value = 0.140) are candidate variable for multivariate analysis with P value < 0.2 (Table 4).

**Table 4:** A bivariate analysis table shows the correlation between socio-demographic characteristics with maternal and child health continuum of care in Gode district, Somali regional State;2022

Variables	Category	MCH continuum of care		COR ((95% CI)	*P*-value
		Yes	No		
Age of women in years	15–24	6(6.98%)	80(93.02%)	1	
	25–34	32(15.24%)	178(84.76%)	2.4(0.96–5.96)	0.06
	35–49	24(14.72%)	139(85.28%)	2.3(0.9–5.86)	0.081
Ethnicity	Somali	59(13%)	395(87%)	0.1(0.02–0.6)	0.012
	Others^2^	3(60%)	2(40%)	1	
Religion	Muslim	59(95.16%)	395(99.5%)	0.3(0.03–3.5)	0.340
	Others^3^	3(4.84%)	2(0.5%)	1	
Marital Status	Married	57(13.16%)	376(86.84%)	0.6(0.2–1.8)	0.383
	Others^4^	5(19.23%)	21(80.77%)	1	
Educational Status	No Education (Unable to read and write)	24(38.71%)	212(53.4%)	1	
	Primary Education (1–8)	32(51.61%)	141(35.52%)	2.0(1.1–3.5)	0.017
	Secondary Education and above	6(9.68%	44(11.08%)	1.2(0.5–3.1)	0.702
Maternal Occupation	Government Employee	21(33.87%)	95(23.93%)	1.6(0.9–2.9)	0.096
	others^5^	41(12%)	302(88%)	1	
Husband Occupation	Government Employee	29(48.33%)	138(35.29%)	1.71(0.99–2.96)	0.053
	Others	31(10.9 2%)	253(89.08%)	1	
Head of the household	Husband	57(91.94%)	366(92.19)	0.96(0.36–2.59)	0.944
	Wife	5(8.06%)	31(7.81%)	1	
Bread Winner	Husband	31(50%)	276(69.52%)	1	
	Wife	7(11.29%)	31(7.81%)	2.0(0.8–4.9)	0.128
	Both	24(38.71%)	90(22.67%)	2.4(1.3–4.3)	0.004
Family Size	Less than five	28(45.16%)	218(54.91%)	1	
	Six up to Ten	30(48.39%)	156(39.29%)	1.5(0.9–2.6)	0.154
	Eleven up to Fifteen	4(6.45%)	23(5.79%)	1.4(0.4–4.2)	0.600
Under five children	One	13(20.97%)	118(29.72%)	1	
	Two	34(54.84%)	228(57.43%)	1.4(0.7–2.7)	0.381
	Three	15(24.19%)	51(12.85%)	2.7(1.2-6.0)	0.018
Family income (In Birr)	< 5,000	15(24.19%)	132(33.25%)	1	
	5,000–10,000	27(43.55%)	143(36.02%)	1.7(0.8–3.3)	0.140
	10,000–20,000	15(24.19%)	96(24.18%)	1.4(0.6–2.9)	0.413
	> 20,000	5(8.06%)	26(6.55%)	1.7(0.6–5.1)	0.347

### Pregnancy related characteristics with MCHCC

Crude analysis of socio-demographic characteristics through bivariate logistic regression showed that types of pregnancy (Planned) (COR = 1.9,95% CI = 1.0–4.0, p-value = 0.067) is a candidate variable to multivariate analysis with p value < 0.2 (Table 5).

**Table 5:** A bivariate analysis table shows the correlation between pregnancy related characteristics with maternal and child health continuum of care in Gode district, Somali regional State;2022

Variables	Category	MCH continuum of care		COR (95%CI)	P value
		Yes	No		
Appropriate time to begin ANC (in weeks)	Before 16 weeks	22(15.49%)	120(84.51%)	1.3(0.7–2.2)	0.406
	16 weeks and more	40(12.62%)	277(87.38%)	1	
Gravidity	< 5	35(12.87%)	237(87.13%)	1	
	≥ 5	27(14.44%)	160(85.56%)	1.1(0.7-2.0)	0.629
Parity	Less than five	43(14.1%)	262(85.9%)	1	
	Five and more	19(12.34%)	135(87.66%)	0.9(0.5–1.5)	0.603
Types of pregnancy	Planned	52(15.25%)	289(84.75%)	1.9(1.0–4.0)	0.067
	Unplanned	10(8.47%)	108(91.53%)	1	
Need of sex preference of HCP	Male	17(12.23%)	122(87.77%)	1	
	Female	45(14.06%)	275(85.94%)	1.3(0.7–2.4)	0.384

### Community related factors with MCHCC

Crude analysis of community related factors through bivariate logistic regression showed that residence (rural) (COR = 2.1,95% CI = 1.0–3.0, p-value = 0.039) and perceived time to reach health facility (< 30 min) (COR = 2.8,95% CI = 1.2–6.3, p-value = 0.014) is a candidate variable to multivariate analysis with p value < 0.2 (Table 6).

**Table 6:** A bivariate analysis table shows the correlation between community related factor with maternal and child health continuum of care in Gode district, Somali regional State;2022

Variables	Category	MCH continuum of care		COR with CI	P value
		Yes	No		
Residence	Urban	28(10.7%)	235(89.4%)	1	
	Rural	34(17.4%)	162(82.7%)	2.1(1.0–3.0)	0.039
Distance to the health facility (km)	Less than five	43(12.5%)	301(87.5%)	1	
	Five to nine	11(17.2%)	53(82.8%)	1.5(0.7–2.99)	0.312
	Ten and more	8(15.7%)	43(84.3%)	1.3(0.6–2.95)	0.528
Perceived time to reach health facility	< 30 min	55(15.8%)	293(84.2%)	2.8(1.2–6.3)	0.014
	≥ 30 min	7(6.3%)	104(93.7%)	1	

### Institutional related factors with MCHCC

Crude analysis of institutional related factors through bivariate logistic regression showed that health professional behavior (good) (COR = 0.6,95% CI = 0.3-1.0, p-value = 0.06), Drug and medical equipment access(no) (COR = 2.0,95% CI = 1.2–3.5, p-value = 0.012) and Maternity waiting room in nearby health facility (COR = 1.5,95% CI = 0.9–2.7, p-value = 0.129) are a candidate variable to multivariate analysis with p value < 0.2 (Table 7).

**Table 7:** A bivariate analysis table shows the correlation between Institutional related factors with maternal and child health continuum of care in Gode district, Somali regional State;2022

Variables	Category	MCH continuum of care		COR (95% CI)	P-value
		Yes	No		
Health Professional Behavior	Good	37(11.6%)	283(88.4%)	0.6(0.34–1.04)	0.06
	Not Good	25(18.0%)	114(82.0%)	1	
Drug and medical equipment access	Yes	35(10.9%)	287(89.1%)	1	
	No	27(19.7%)	110(80.3%)	2.0(1.2–3.5)	0.012
Maternity waiting room in nearby health facility	Yes	40(15.7%)	215(84.3%)	1.5(0.9–2.7	0.129
	No	22(10.8%)	182(89.2%)	1	

### Multivariate logistic regression

Multivariable logistic regression was performed to control the confounding effect of variables. Accordingly, Husband occupation (Government employee) [AOR = 2.3, 95%CI: (1.2–4.7)] and perceived time to reach health facility (less than 30 min) [AOR = 2.96, 95%CI: (1.2–7.5)] were factors showing significant association with maternal and child health continuum of care among mothers in Gode district, Somali regional State;2022 at P-value ≤ 0.05. The odds of maternal and child health continuum of care among mothers had husband employed in government organization 2.3 times [AOR = 2.3, 95%CI: (1.2–4.7)] higher than others. The odds of maternal and child health continuum of care among mothers perceived that distance to reach health facility less than 30 min 2.96 times [AOR = 2.96, 95%CI: (1.2–7.5)] higher than those perceived as greater than 30 min (Table 8).

**Table 8:** A multivariate analysis on maternal and child health continuum of care in Gode district, Somali regional State;2022

Variables	Category	MCH continuum of care		COR (95% CI)	AOR (95% CI)	P-value
		Yes	No			
Age of women in years	15–24	6(7%)	80(93.0%)	1		
	25–34	32(15%)	178(84.8%)	2.4(0.96–5.96)	1.9(0.7–5.3)	0.226
	35–49	24(14.7%)	139(85.3%)	2.3(0.9–5.86)	1.9(0.6–6.5)	0.282
Ethnicity	Somali	59(13%)	395(87%)	0.1(0.02–0.6)	0.2(0.02–1.2)	0.079
	Others	3(60%)	2(40%)	1	1	
Educational Status	No Education (Unable to read and write)	24(38.7%)	212(53.4%)	1	1	
	Primary Education (1–8)	32(51.6%)	141(35.5%)	2.0(1.1–3.5)	1.5(0.8–3.1)	0.244
	Secondary Education and above	6(9.7%)	44(11.1%)	1.2(0.5–3.1)	1.1(0.3–3.5)	0.896
Husband occupation	Government Employee	29(48.3%)	138(35.3%)	1.71(0.99–2.96)	2.3(1.2–4.7)	**0.016**
	Others	31(10.9%)	253(89.1%)	1	1	
Head of the household	Husband	57(91.9%)	366(92.2)	0.96(0.36–2.59)	3.3(0.3–32.2)	0.306
	Wife	5(8.1%)	31(7.8%)	1	1	
Bread Winner	Husband	31(50%)	276(69.5%)	1	1	
	Wife	7(11.3%)	31(7.8%)	2.0(0.8–4.9)	5.6(0.7–41.4)	0.094
	Both	24(38.7%)	90(22.7%)	2.4(1.3–4.3)	1.9(0.9–3.9)	0.078
Family Size	Less than five	28(45.2%)	218(54.9%)	1		
	Six up to Ten	30(48.4%)	156(39.3%)	1.5(0.9–2.6)	1.5(0.7–3.3)	0.277
	Eleven up to Fifteen	4(6.5%)	23(5.8%)	1.4(0.4–4.2)	2.4(0.6–10.5)	0.243
No. of Under five children	One	13(21.0%)	118(29.7%)	1	1	
	Two	34(54.9%)	228(57.4%)	1.4(0.7–2.7)	1.2(0.5–2.5)	0.702
	Three	15(24.2%)	51(12.9%)	2.7(1.2-6.0)	1.7(0.7–4.4)	0.266
Types of pregnancy	Planned	52(15.3%)	289(84.8%)	1.9(1.0–4.0)	1.2(0.5-3.0)	0.626
	Unplanned	10(8.5%)	108(91.5%)	1	1	
Residence	Urban	28(10.7%)	235(89.4%)	1	1	
	Rural	34(17.4%)	162(82.7%)	2.1(1.0–3.0)	0.6(0.25–1.23)	
Perceived time to reach health facility	< 30 min	55(15.8%)	293(84.2%)	2.8(1.2–6.3)	2.96(1.17–7.5)	**0.022**
	≥ 30 min	7(6.3%)	104(93.7%)	1	1	

## Disscusion

This study conducted in Gode district, Ethiopia, yielded mixed findings regarding maternal healthcare utilization. While a high proportion of participants (90.9%) reported attending at least one antenatal care (ANC) visit during a prior pregnancy, suggesting some awareness of its significance, only 19.2% received the recommended minimum of four visits. This falls short of both the national average of 44% reported in the latest Ethiopian Demographic and Health Survey (EDHS) and the considerably higher rates observed in urban centers like Addis Ababa (84%). Notably, even within the Somali region, Gode exhibits the lowest recorded rate (19.2% compared to the regional average of 23.9%) [9]. This disparity underscores the persistent challenges in achieving equitable access to high-quality maternal healthcare services in rural Ethiopia. Geographical barriers likely play a significant role in Gode, with limited healthcare facilities and substantial travel distances for expecting mothers. These geographical constraints can be further compounded by cultural factors and economic limitations that restrict women’s ability to access adequate ANC care. Additionally, a potential knowledge gap regarding the benefits of ANC, particularly among first-time mothers, could further hinder utilization.

However, the study found positive trends in facility deliveries (86.7%) and postnatal care utilization (81.9%). This suggests a potential government effort in expanding access to delivery care and the value of institutional deliveries. Similarly, postnatal care utilization (75.4% within two days) was higher than national averages (33.8% within 48 h) [29]. This might be due to Ethiopian government initiatives promoting postnatal care services.

This study finding showed that the prevalence of MCH continuum of care was 13.5% (10.7-17.0%). This finding is lower than a study finding in different part of Ethiopia such as, Wayu district 16.1% and Debrebrhan town 37.2% North west Ethiopia 21.60% [30–32]. However, this finding is higher than a study finding in Ethiopia (2%) [33].It is also higher than findings of different part of the world Cambodia and Korea, which were 5.0% [34] and 6.8% [35] respectively. The probable reason for such variation could be different access to health care services, economic status, and education.

The odds of maternal and child health continuum of care among mothers had husband employed in government organization 2.3 times higher than others. Occupational status being other than a farmer, clinical/sales employed and not involved in agricultural activities are more likely to get the maternal, newborn and child health continuum of care respectively compared to their counterparts in Studies finding in Lao PDR in rural Khammouane, Nigeria, and Ethiopia [19, 36, 37]. But in Pakistan, occupational status was not a significant factor [17]. A husband’s occupation can have a significant impact on the maternal and child health continuum of care. Spousal employment in the government sector, characterized by typically stable salaries and comprehensive benefit packages, might be associated with a higher likelihood of women accessing prenatal care, delivering at healthcare facilities, and ensuring their children’s complete vaccination schedules.

The odds of maternal and child health continuum of care among mothers perceived that distance to reach health facility less than 30 min 3.0 times higher than those perceived as greater than 30 min. It is similar with study finding on Wayu district and northwest Ethiopia [32, 38]. Long distances to health facilities can translate to significant travel times, especially in rural areas with limited public transportation options. This can be a major deterrent, particularly for pregnant women or those with young children.

### Strength and limitation

This investigation assesses service utilization within the maternal and child health continuum in a pastoral setting. However, the generalizability of these findings to the entire nation may be limited due to the study being confined to a single district. It is important to acknowledge that recall bias could be a potential limitation.

## Conclusion

This study in Gode district, Somali Region, Ethiopia (2022) identified a relatively low prevalence of complete utilization of the maternal and child health (MCH) continuum of care. Despite a high proportion of participants having at least one antenatal care (ANC) visit during a previous pregnancy, only less than one fifth received the recommended four or more ANC visits. However, facility deliveries and postnatal care utilization were encouraging.

A study suggests that husband occupation (government employment) and perceived time to reach health facility (less than 30 min) appears to be a crucial factor influencing MCH service utilization.

### Recommendation

Invest in healthcare infrastructure, especially in remote areas, to make services more accessible. Strengthen existing programs and facilities to keep delivery and postnatal care utilization rates high. Explore possibilities of integrating ANC visits with delivery and postnatal care services to create a more cohesive MCH experience.

## Data Availability

The datasets generated and/or analyzed during the current study are available from the corresponding author on reasonable request.

## Footnotes

1 NGO.

2 Amhara and Oromo

3 Orthodox and protestant

4 Widowed & divorced

5 Farmer, Marchant & Housewife

## Abbreviations

ANC: Antenatal Care
AOR: Adjusted Odd Ratio
BPCR: Birth Preparedness and Complication Readiness
CoC: Continuum of Care
COR: Crude Odd Ratio
CSA: Central Statistics Agency
FP: Family Planning
HIV: Human Immunodeficiency Virus
MNCH: Maternal Neonatal and Child Health
MCH: Maternal and Child Health
PNC: Post Natal Care
SBA: Skilled Birth Attendance
SPSS: Statistical Package for Social Sciences
WHO: World Health Organization
